# Human liver infiltrating γδ T cells are composed of clonally expanded circulating and tissue-resident populations

**DOI:** 10.1016/j.jhep.2018.05.007

**Published:** 2018-09

**Authors:** Stuart Hunter, Carrie R. Willcox, Martin S. Davey, Sofya A. Kasatskaya, Hannah C. Jeffery, Dmitriy M. Chudakov, Ye H. Oo, Benjamin E. Willcox

**Affiliations:** 1 Cancer Immunology and Immunotherapy Centre, Institute of Immunology and Immunotherapy, University of Birmingham, Edgbaston, Birmingham B15 2TT, United Kingdom; 2 Centre for Liver Research and National Institute for Health Research (NIHR) Birmingham Biomedical Research Centre, Institute of Immunology & Immunotherapy, University of Birmingham, United Kingdom; 3 Shemyakin-Ovchinnikov Institute of Bioorganic Chemistry, Russian Academy of Science, Moscow, Russia; 4 Skolkovo Institute of Science and Technology, Moscow, Russia; 5 Central European Institute of Technology, Masaryk University, Brno, Czech Republic; 6 Pirogov Russian National Research Medical University, Moscow, Russia; 7 University Hospital of Birmingham NHS Foundation Trust, United Kingdom

**Keywords:** Gamma delta T cells, T cell receptor, Liver immune surveillance, Liver-resident T cells, Human liver, Immunological memory

## Abstract

**Background & Aims:**

γδ T cells comprise a substantial proportion of tissue-associated lymphocytes. However, our current understanding of human γδ T cells is primarily based on peripheral blood subsets, while the immunobiology of tissue-associated subsets remains largely unclear. Therefore, we aimed to elucidate the T cell receptor (TCR) diversity, immunophenotype and function of γδ T cells in the human liver.

**Methods:**

We characterised the TCR repertoire, immunophenotype and function of human liver infiltrating γδ T cells, by TCR sequencing analysis, flow cytometry, *in situ* hybridisation and immunohistochemistry. We focussed on the predominant tissue-associated Vδ2^−^ γδ subset, which is implicated in liver immunopathology.

**Results:**

Intrahepatic Vδ2^−^ γδ T cells were highly clonally focussed, with single expanded clonotypes featuring complex, private TCR rearrangements frequently dominating the compartment. Such T cells were predominantly CD27^lo/−^ effector lymphocytes, whereas naïve CD27^hi^, TCR-diverse populations present in matched blood were generally absent in the liver. Furthermore, while a CD45RA^hi^ Vδ2^−^ γδ effector subset present in both liver and peripheral blood contained overlapping TCR clonotypes, the liver Vδ2^−^ γδ T cell pool also included a phenotypically distinct CD45RA^lo^ effector compartment that was enriched for expression of the tissue tropism marker CD69, the hepatic homing chemokine receptors CXCR3 and CXCR6, and liver-restricted TCR clonotypes, suggestive of intrahepatic tissue residency. Liver infiltrating Vδ2^−^ γδ cells were capable of polyfunctional cytokine secretion, and unlike peripheral blood subsets, were responsive to both TCR and innate stimuli.

**Conclusion:**

These findings suggest that the ability of Vδ2^−^ γδ T cells to undergo clonotypic expansion and differentiation is crucial in permitting access to solid tissues, such as the liver, which results in functionally distinct peripheral and liver-resident memory γδ T cell subsets. They also highlight the inherent functional plasticity within the Vδ2^−^ γδ T cell compartment and provide information that could be used for the design of cellular therapies that suppress liver inflammation or combat liver cancer.

**Lay summary:**

γδ T cells are frequently enriched in many solid tissues, however the immunobiology of such tissue-associated subsets in humans has remained unclear. We show that intrahepatic γδ T cells are enriched for clonally expanded effector T cells, whereas naïve γδ T cells are largely excluded. Moreover, whereas a distinct proportion of circulating T cell clonotypes was present in both the liver tissue and peripheral blood, a functionally and clonotypically distinct population of liver-resident γδ T cells was also evident. Our findings suggest that factors triggering γδ T cell clonal selection and differentiation, such as infection, can drive enrichment of γδ T cells into liver tissue, allowing the development of functionally distinct tissue-restricted memory populations specialised in local hepatic immunosurveillance.

## Highlights

• Intrahepatic Vδ2^neg^ γδ T cells are clonally focussed and feature private TCR rearrangements.
• Effector CD27^lo/neg^ Vδ1^+^ T cells are enriched in liver, but naïve CD27^hi^ cells are absent.
• A subset of Vδ1^+^ T cells is distinct from those in blood and may be liver tissue resident.
• Liver Vδ1^+^ γδ T cells are polyfunctional and respond to both TCR and innate stimuli.

## Graphical abstract

**Figure:**
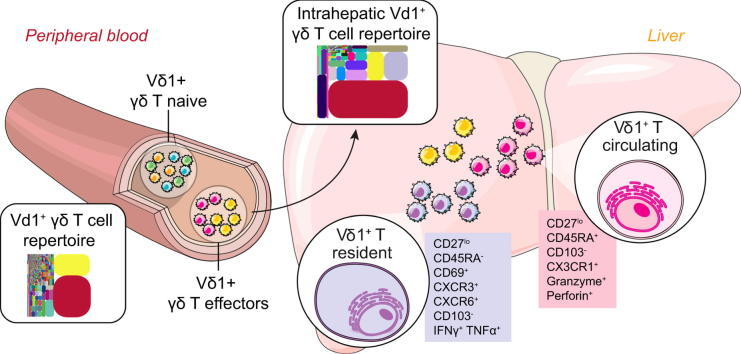

## Introduction

γδ T cells are unconventional lymphocytes enriched in solid tissues, where they are thought to play critical roles in immunosurveillance.^1^ Studies of mouse tissue-associated γδ subsets suggest γδ T cell function can be predominantly innate-like, involving semi-invariant T cell subsets that enable fast response kinetics without a requirement for clonal selection and differentiation.[2], [3], [4], [5] This role may allow for rapid ‘lymphoid stress surveillance’, limiting damage to host tissues in the face of microbial or non-microbial challenges, prior to full activation of adaptive immunity.[4], [6] As such, γδ T cells may critically complement the contributions of tissue-resident αβ subsets, which provide an augmented adaptive response to infections re-encountered at body surfaces,^7^ potentially explaining the retention of γδ T cells alongside the αβ T cell and B cell lineage over 450 million years of vertebrate evolution.^8^

In contrast, the paradigms underlying human γδ T cell immunobiology are far from clear. In humans, the peripheral blood is dominated by the Vδ2^+^/Vγ9^+^ T cell subset, polyclonally activated by bacterial^9^ and endogenous phospho-antigens,^10^ arguably conforming to an innate-like paradigm.^11^ In contrast, human solid tissues are enriched for Vδ2^−^ γδ T cells, of which the Vδ1^+^ subset is the most prevalent. It is far less clear if this dominant human tissue-associated subset also adopts an innate-like biology. Indeed, Vδ2^−^ T cells have been linked to recognition of a diverse range of ligands including to date Endothelial Protein C Receptor,^12^ CD1 molecules,^13^ Annexin-A2,^14^ and even phycoerythrin.^15^ Moreover, recent data have provided strong evidence that Vδ1^+^ cells display an unconventional adaptive biology, undergoing clonal selection and differentiation from a naïve T cell receptor (TCR)-diverse precursor pool,^16^ with viral infection one trigger driving expansion.^17^ However, such studies have focussed on the subset of Vδ2^−^ γδ T cells that are retained in peripheral blood. To date, the immunobiology of human tissue-associated γδ T cells remains relatively unstudied, despite the Vδ2^−^ T cell subset representing a considerable proportion of the total T cell infiltration in many human solid tissues, including gut,^2^ lung^18^ and liver.^19^

To shed light on the function of tissue-associated γδ T cells and how this relates to peripheral subsets, we characterised human intrahepatic Vδ2^−^ T cells. The liver is a site of considerable blood flow, receiving 75% of the total blood in the body every 2 h, with a third of this originating directly from the antigen-rich gut via the portal vein. In addition to providing a generally immunosuppressive microenvironment to facilitate tolerization of T cells toward non-pathogenic antigens present in the portal blood flow, the liver is also home to a large population of innate lymphoid cells, including natural killer (NK) cells, invariant natural killer T (iNKT) cells, mucosal associated invariant T (MAIT) cells^20^ and γδ T cells,^19^ in addition to CD8^+^ cytotoxic T cells.^21^ This enrichment is believed to balance the need for tolerization with a requirement for rapid identification and elimination of potentially harmful pathogenic entities, for example via pathogen associated molecular pattern receptors and semi-invariant T cell populations.^22^ To shed light on the immunobiology of γδ T cells in this context we exploited next generation sequencing (NGS) approaches, allowing us to probe the TCR repertoire, in parallel with immunophenotype, and function.

Our study is the first to define the interconnected clonotypic, phenotypic and functional features of human tissue-associated γδ T cells. The findings suggest that the liver selectively retains Vδ2^−^ T cells that are clonally expanded and adopt an effector phenotype, and which include a subset containing liver-restricted clonotypes that is phenotypically and functionally distinct from those present in peripheral blood.

## Material and methods

### Ethical approval and samples

**Fig. 1:**
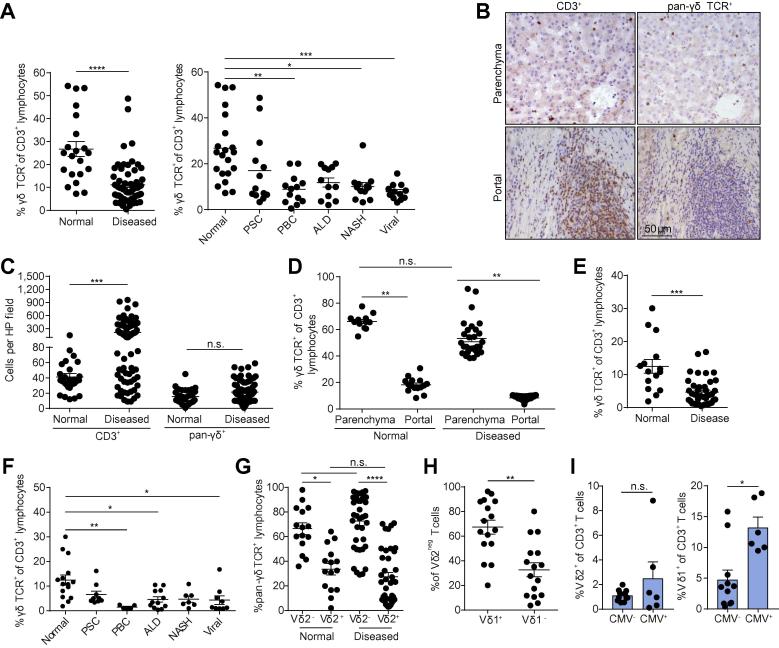
**Normal liver parenchyma is enriched for γδ T cells.** (A) Comparison of γδ TCR^+^ proportion of CD3^+^ T cells identified by IHC in normal (n = 21) and diseased (n = 62) liver tissue (left) and in CD3^+^ T cells identified by IHC in normal (n = 21), PSC (n = 13), PBC (n = 13), ALD (n = 12), NASH (n = 12) and viral hepatitis (n = 12) liver tissue (right). (B) Representative staining for CD3^+^ (left) and γδ TCR^+^ (right) cells on sequential FFPE sections from NASH liver tissue viewed at 40× magnification. (C) Densities of CD3^+^ and γδ TCR^+^ cells in normal (n = 21) and diseased (n = 62) liver tissue. (D) Comparison of the γδ TCR^+^ proportion of CD3^+^ T cells identified by IHC in parenchymal and portal areas of normal (n = 15) and diseased (n = 30) liver tissue. (E) Comparison of the γδ TCR^+^ proportion of CD3^+^ T cells identified by flow cytometry in normal (n = 15) and diseased (n = 42) liver cell suspensions. (F) Comparison of the γδ TCR^+^ proportion of CD3^+^ T cells identified by flow cytometry in normal (n = 15) and diseased liver cell suspensions of various aetiologies. (G) Comparison of Vδ2^+^ and Vδ2^−^ proportions in γδ TCR^+^ cells identified by flow cytometry from normal (n = 15) and diseased (n = 42) liver cell suspensions. (H) Comparison of Vδ1^+^ and Vδ1^−^ proportions in Vδ2^−^ cells from liver cell suspensions (n = 16). (I) Comparison of Vδ2^+^ (left) and Vδ1^+^ (right) proportion of CD3^+^ T cells in CMV^−^ (n = 11) and CMV^+^ donors (n = 6) from diseased livers. Error bars indicate mean ± SEM; data analysed by Kruskal-Wallis ANOVA with Dunn’s post-test comparisons, n.s. *p* >0.05, **p <0.01, ***p <0.001 and ****p <0.0001. ALD, alcoholic liver disease; CMV, cytomegalovirus; FFPE, formalin-fixed paraffin embedded; IHC, immunohistochemistry; NASH, non-alcoholic steatohepatitis; PBC, primary biliary cholangitis; PSC, primary sclerosing cholangitis; TCR, T cell receptor. (This figure appears in colour on the web.)

Explanted diseased liver tissue and matched blood were obtained from patients who underwent liver transplantation for end-stage liver diseases including primary sclerosing cholangitis (PSC), primary biliary cholangitis (PBC), alcoholic liver disease (ALD), non-alcoholic steatohepatitis (NASH), hepatitis C virus (HCV) and hepatitis B virus (HBV) (Local Research Ethics Committee reference No. 98/CA5192) or normal liver samples from donor liver tissue surplus to clinical requirements (Local Research Ethics Committee reference No. 06/Q2708/11). Unless otherwise stated (see Fig. 1), all diseased liver tissue analysed was from HCV/HBV-negative donors, and were non-cancerous. Normal liver tissue donors had no known prior history of liver disease or HCV/HBV infection. All diseased livers were Child C decompensated. Adult peripheral blood was obtained from consenting healthy donors (protocol approved by the NRES Committee West Midlands ethical board; REC reference 14/WM/1254).

### T cell isolation, culture and activation

Human liver infiltrating lymphocytes were isolated from fresh liver tissue as described previously.^20^ A whole slice of liver was processed, thereby reducing any effects of heterogeneous disease localisation. Briefly, explanted liver tissue was diced into 5 mm^3^ cubes, washed with Phosphate Buffered Saline (PBS), and then homogenised in a Seward stomacher 400 circulator (260 rpm, 5 min). The homogenate was filtered through fine (63 µm) mesh (John Staniar and Co, Manchester, UK) and the lymphocytes were isolated by density gradient separation using Lympholyte (VH Bio, Gateshead, UK) at 800×*g* for 20 min. The lymphocyte layer was collected and washed with PBS. Cell viability was assessed by trypan blue exclusion. Peripheral blood mononuclear cells (PBMCs) were isolated from heparinised venous blood by lymphoprep© (Stem Cell Technologies) density gradient centrifugation as per the manufacturer’s instructions. The cell culture medium used throughout this study was RPMI-1640 medium (Invitrogen) supplemented with 2 mM l-glutamine, 1% sodium pyruvate, 50 μg/ml penicillin/streptomycin (Invitrogen) and 10% foetal calf serum (Sigma).

### Antibodies and flow cytometry

For total and single-cell sorting of Vδ2^−^ and Vδ1^+^ γδ T populations, PBMC were labelled with anti-CD3 (UCHT1; BioLegend), TCR γδ (BW242/412), TCR Vδ2 (123R3) or TCR Vδ1 (REA173); all Miltenyi, CD27 (M-T271), CD45RA (HI100); BioLegend, and populations were sorted on a MoFlo Astrios (Beckman Coulter) or ARIA III Fusion (BD). For repertoire analysis, Vδ2^−^ T cell populations were sorted directly into RNAlater (Sigma). For phenotypic analysis, freshly isolated or frozen PBMCs, or cultured cells were labelled with Zombie Aqua viability dye (BioLegend), and then subsequently stained for cell surface antigens with antibodies directed against CD3 BV421 (UCHT-1, 1:100), CD8 BV650 (SK1; 1:200), CD45RA PeCy7 (HI100; 1:200), CD27 PE/Dazzle 594 (M-T271; 1:200), CCR7 AF647 (G043H7; 1:100), CD62L APC-Cy7 (DREG-56; 1:100), CD28 PE (28.2; 1:80), CD16 PE-Cy7 (3G8; 1:100), CD69 BV605 (FN50; 1:100), CD25 BV421 (2A3; 1:100), CD54 BV421 (HA58; 1:100), TCR Vδ2 PE (B6; 1:100), TCR γδ PE Cy7 (B1; 1:100), TCR αβ PE (IP26; 1:50), CXCR3 PE (G025H7); all BioLegend. CXCR6 PE (56811/FAB699P; 1:20) from R&D Systems. Mouse anti-human CX_3_CR1-PE (2A9–1; 1:20), CD69 PE (FN50; 1:50) from Immunotools. Mouse anti-human CD127 APC (IM1980U; 1:20), TCR γδ PE Cy7 (IMMU510; 1:200), TCR Vδ3 FITC and TCR Vγ9 PE Cy5 (IMMU360; 1:400); Beckman Coulter. TCR Vδ1 PE (TS8.2; 1:100); Fisher Scientific. TCR Vδ1 PE and FITC (REA173; 1:100) and TCR Vδ2 APC (123R3; 1:200); Miltenyi Biotec. For intracellular staining, after surface antibody staining, cells were fixed in Foxp3/Transcription factor fix/perm buffer (eBioscience) and stained in permeabilization buffer (eBioscience) with antibodies directed against Granzyme A FITC (CBO9; 1:100), Granzyme B APC (GB11; 1:100) and Perforin BV421 (B-D48; 1:80); all BioLegend. For intracellular cytokine staining, antibodies used were interferon-γ (IFNγ) BV421 (340449; 1:200), tumour necrosis factor alpha (TNFα) PE (554512; 1:200); BD Pharmingen, Cells were acquired on a CyAn ADP (Beckman Coulter), LSR II or LSR Fortessa X20 (BD) and data analysed with FlowJo V10.2 (TreeStar) or Summit 4.3 software (Dako Cytomation).

### Immunohistochemistry and ***in situ*** hybridisation

Immunohistochemistry was performed using formalin fixed paraffin embedded (FFPE) sections using standard approaches. In summary, sections were de-paraffinized, endogenous peroxidase activity was quenched using 0.3% hydrogen peroxide (Sigma Aldrich) in methanol for 20 min, and antigen retrieval carried out, involving boiling sections in 1% EDTA solution for 15 min. After washing and blocking steps, sections were incubated for 1 h in primary antibody (goat polyclonal – anti-human pan-VγVδ (50 μg/ ml, A-20, Santa Cruz Biotechnology, Santa Cruz, USA) or rabbit polyclonal – anti-human CD3 (2 μg/ml, ab5690, Abcam, Cambridge, UK) or relevant IgG1 isotype control) diluted in PBS. After washing, sections were incubated with HRP-linked anti-goat or anti-rabbit secondary antibody (Vector Labs Laboratories) for 30 min at room temperature. Following washing, sections were developed using ImmPACTTM DAB reagent (Vector Laboratories). Excess DAB was then removed by rinsing and sections were counterstained with Mayer’s haematoxylin solution (Leica Biosystems). Once dry, slides were mounted using DPX (Cellpath, Newtown Powys, UK) and imaged on a Zeiss Axioskop 40 Microscope. Regions of parenchymal and portal tract tissue were identified and numbers of CD3+ or γδ-TCR+ cells were counted per region identified, with five high power fields, selected at random, scored for each section.

For *in situ* hybridisation, TCR chain-specific localisation of gamma delta TCR+ cells was performed using two protocols, either the ViewRNA™ ISH Tissue 2-Plex Assay developed by Affymetrix and performed manually, or the RNAscope® 2.5 LS Duplex Assay (ACD. For both protocols, liver slices were cut and immediately fixed in formalin for 24–48 h prior to being embedded in paraffin and mounted. Immediately after which the assay slides were baked at 60 °C for 1 h to immobilise the sections.

### TCR repertoire analysis

RNA was purified from sorted cells (intrahepatic Vδ2^−^ T cells: 8,000–50,000 cells) protected in RNA*later* (Sigma Aldrich) using an RNAmicro plus kit (Qiagen) according to the manufacturer’s instructions. For high throughput deep sequencing of γδ TCRs, we used amplicon rescued multiplex (ARM)-PCR and a MiSeq (illumina) next generation sequencer to analyse all sorted Vδ2^−^ T cell populations. Following initial first-round RT-PCR using high concentrations of gene-specific primers, universal primers were used for the exponential phase of amplification (Patent: WO2009137255A2), allowing deep, quantitative and non-biased amplification of TCRγ and TCRδ sequences. All cDNA synthesis, amplification, NGS library preparation and sequencing were performed by iRepertoire, Inc. (Huntsville, USA).

### Single-cell TCR sequencing

PBMCs were labelled as described above and Vδ1^+^ T cells were single-cell sorted directly into individual wells in a 96 well plate containing 2 µl of Superscript VILO cDNA synthesis kit reaction mix (ThermoFisher) containing 0.1% Triton X-100, and incubated according to manufacturer’s instructions. TCRγ and TCRδ cDNAs were amplified by two rounds of nested PCR using GoTaq mastermix (Promega) and primers for or Vδ1, CAAGCCCAGTCATCAGTATCC (external) and CAACTTCCCAGCAAAGAGATG (internal); for Cδ GCAGGATCAAACTCTGTTATCTTC (external) and TCCTTCACCAGACAAGCGAC (internal); for Vδ3, GGCACGCTGTGTGACAAA (external) and CTGCTCTGCACTTACGACACTG (internal); for Vγ1–8 CTGGTACCTACACCAGGAGGGGAAGG (external) and TGTGTTGGAATCAGGAVTCAG (internal); for Vγ9 AGAGAGACCTGGTGAAGTCATACA (external) and GGTGGATAGGATACCTGAAACG (internal) and for Cγ CTGACGATACATCTGTGTTCTTTG (external) and AATCGTGTTGCTCTTCTTTTCTT (internal). PCR products were separated on 1.2% agarose gels, and products of successful reactions were incubated with ExoSAP-IT PCR cleanup enzyme (Affymetrix) before sequencing with BigDye Terminator v3.1 (Applied Biosystems) following manufacturer’s instructions and running on an ABI 3730 capillary sequencer (Functional Genomics Facility, University of Birmingham).

### TCR repertoire data analysis

Sequences data was error corrected and V, D and J gene usage and complementarity-determining region 3 (CDR3) sequences were identified and assigned, and tree maps generated using iRweb tools (iRepertoire, Inc, Huntsville, AL, USA). Tree maps show each unique CDR3 as a coloured rectangle, the size of each rectangle corresponds to each CDR3s abundance within the repertoire and the positioning is determined by the V region usage. For more detailed analysis and error correction of the TCR repertoire, datasets were processed using the MiXCR software package to further correct for PCR and sequencing errors. Diversity metrics, clonotype overlap and gene usage were plotted in R, by VDJTools.

### TCR sequence analyses

The CDR3 length was defined as the number of amino acids between the second cysteine of the V region and the phenylalanine of the J region, according to IMGT. N and P nucleotides were identified using the IMGT Junction Analysis tool.

### Statistical analysis

Tabulated data were analysed in Graphpad PRISM 7 (Graphpad Software Inc). Each data set was assessed for normality using Shapiro-Wilk normality test. Differences between columns were analysed by two-tailed Student’s *t* tests for normally distributed data and Mann-Whitney for non-parametric data. Differences between groups were analysed using one-way ANOVA with Tukey’s post-tests for normally distributed data or with Kruskal-Wallis with Tukey’s post-tests for non-parametric data and RM two-way ANOVA with Tukey’s post-tests was used when comparing groups with independent variables. ∗*p* <0.05, ∗∗*p* <0.01, ∗∗∗*p* <0.001 and ∗∗∗∗*p* <0.0001.

### Data availability

The sequence data that support the findings of this study have been deposited in the NIH NCBI sequence read archive database with the primary accession code SRP113556 and SRP096009, for γδ TCR repertoires. For more detailed metadata relating to individual samples please contact the authors.

For further details regarding the materials used, please refer to the CTAT table and Supplementary information.

## Results

Human Vδ2^−^ γδ T cell populations are reportedly tissue tropic in nature, with enrichment of this compartment previously highlighted in diseased human gut^23^ and liver.^19^ We used immunohistochemistry (IHC) analysis to assess the infiltration and localisation of liver γδ T cells. Firstly, γδ T cells were a significantly enriched proportion of infiltrating CD3^+^ T cells in normal livers compared with livers explanted from patients with chronic liver disease (Fig. 1A). Furthermore, we noted the majority of the infiltrating CD3^+^ T cells were localised to portal areas; however, analysis of sequentially stained sections from normal tissue revealed a high proportion of parenchyma-associated CD3^+^ T cells were γδ TCR^+^ (Fig. 1B). Importantly, while a significant increase in infiltrating CD3^+^ T cells was observed in diseased tissue, γδ T cell numbers did not significantly change, suggesting that disease drives an increased infiltration of total CD3^+^ T cells but not γδ TCR^+^ cell infiltration from the periphery (Fig. 1B, C, Fig. S1A). Further analysis of sequentially stained sections from explanted livers confirmed that γδ TCR^+^ cells were also preferentially associated with the liver parenchyma (Fig. 1D, Fig. S1B). We then examined the TCRδ chain expression of liver infiltrating γδ T cell populations by flow cytometry, in homogenised single-cell suspensions of liver tissue from human explanted livers (Fig. S1C). Consistent with our IHC data, a significantly higher proportion of the CD3^+^ T cell compartment was comprised of γδ T cells in healthy liver tissue compared with disease tissue (Fig. 1E-F), of which the majority were Vδ2^−^ (Fig. 1G), a direct inversion of the predominance of Vδ2^+^ T cells in the peripheral blood.[24], [16] Moreover, the majority of the Vδ2^−^ compartment was made up of Vδ1^+^ γδ T cells (Fig. 1H, Fig. S1D), with the remainder comprised of other undefined Vδ chains. Disease aetiology had no observed impact on this observation (Fig. S1E). Consistent with pan-γδ T cell IHC, infiltration of Vδ1^+^ γδ T cells into liver parenchyma was demonstrated using IHC and *in situ* hybridisation; again, IHC staining of sequential sections suggested a high proportion of parenchyma-associated CD3^+^ T cells were Vδ1^+^ (Fig. S1F). Of note, Vδ1^+^ γδ T cells were significantly enriched as a proportion of intrahepatic T cells in diseased cytomegalovirus (CMV)^+^ liver donors compared with diseased CMV^−^ donors, while Vδ2^+^ T cells were not (Fig. 1I).

**Fig. 2:**
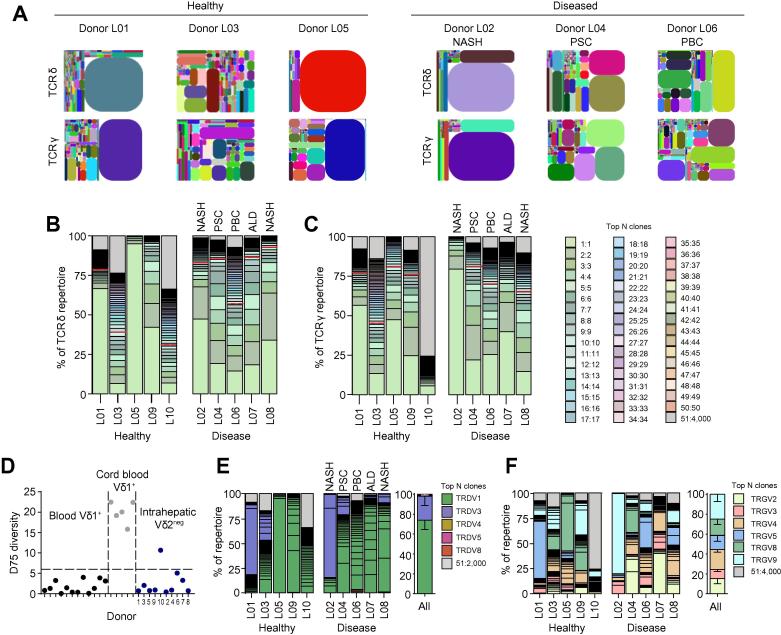
**Intrahepatic Vδ2^−^ γδ T cells are formed of clonally focussed TCR repertoires.** (A) Representative tree maps show CDR3 clonotype usage in relation to repertoire size (each CDR3 colour is chosen randomly and does not match between plots) in TCRδ and TCRγ repertoires from αβ TCR^−^ Vδ2^−^ T cells sorted from normal (n = 5) and diseased livers (n = 5). Proportion of the total (B) TCRδ and (C) TCRγ repertoire occupied by the 50 most prevalent CDR3 sequences from sorted Vδ2^−^ T cells for each sorted liver sample (n = 10). The dashed black line denotes the percentage of the repertoire occupied by the ten most frequent clonotypes. (D) Analysis of inter-donor diversity by D75 (percentage of clonotypes required to occupy 75% of the total TCR repertoire) from TCRδ repertoire analyses from 12 healthy donors (Vδ1^+^), 5 cord blood donors (Vδ1^+^) and 7 liver samples (Vδ2^−^) and lowest quartile range plotted (dashed line). (E) Vδ and (F) Vγ chain usage by the 50 most prevalent γδ TCR CDR3 sequences from sorted Vδ2^−^ T cells from normal and diseased livers with summary plots. Error bars indicate mean ± SEM. CDR3, complementarity determining region 3; TCR, T cell receptor. (This figure appears in colour on the web.)

We next assessed the TCR repertoire of enriched populations of Vδ2^−^ γδ T cells from both healthy and diseased liver tissue by amplicon rescued multiplex (ARM)-PCR and deep sequencing (Fig. S2A). Tree plot and clonotype analysis of Vδ2^−^ TCR repertoires indicated that both healthy and diseased liver tissue was generally dominated by a small number of highly prevalent clonotypes (Fig. 2A–C), with the 10 most prevalent CDR3 sequences accounting for >40% of TCRγ and TCRδ sequences in 9 and 8 out of 10 samples, respectively, and one dominant clone representing >50% in 2 of the 10 TCRγ and TCRδ samples (Fig. 2B-C). Comparison with D75 values obtained from adult and cord blood Vδ1^+^ TCR repertoires placed liver Vδ2^−^ TCR repertoires in a comparable range with other highly focussed γδ TCR repertoires (Fig. 2D). Furthermore, when measuring the number of unique clonotypes detected in the first 10^5^ CDR3 sequences obtained in each sample, an alternative measure of TCR diversity, liver samples displayed a significantly less diverse repertoire than blood γδ TCR repertoires (Fig. S2B). Comparison of Chao1 diversity metrics revealed no difference in the diversity of clonotypes between healthy and diseased liver TCR repertoires (Fig. S2C). Consistent with a broadly similar TCR repertoire in healthy and diseased tissue, comparison of normalised CDR3 lengths from healthy and diseased samples yielded no discernible difference (Fig. S2D). Previous studies have highlighted that peripheral blood Vδ2^−^ TCRγ repertoires contain few shared sequences.[16], [17] We found that liver Vδ2^−^ TCRγ repertoires were in general more private than blood Vδ2^−^ TCRγ repertoires and had very limited shared sequences between unrelated donors (Fig. S2E).

Consistent with flow cytometry analyses (Fig. 1G-H), Vδ chain usage was dominated by Vδ1 (73.96% ±SEM 8.7) and Vδ3 (24.05% ±SEM 9.3) chain usage, with little Vδ4, Vδ5 and Vδ8 usage observed (Fig. 2E). Despite dominant clonotypes, Vγ chain usage was highly heterogeneous, with all coding Vγ chains utilised across our samples (Fig. 2F). Moreover, no significant difference was observed in Vδ or Vγ chain usage between healthy and diseased samples (Fig. 2E-F), consistent with the similar diversity metrics observed in diseased and healthy liver samples. These TCR sequencing data indicate the overwhelming prevalence of Vδ1^+^ TCR sequences in liver tissue, while confirming previous findings demonstrating a relative enrichment of Vδ3^+^ γδ T cells in human liver compared to peripheral blood.^19^ Next, we assessed individual Vδ1^+^ and Vδ3^+^ TCR repertoires for evidence of clonal expansion, initially using accumulated frequency curves to measure the 10 most prevalent clonotypes across all samples (Fig. S2F). These analyses provided evidence for clonal dominance in both liver Vδ1^+^ and Vδ3^+^ TCR repertoires, similar to clonotypically focussed peripheral blood Vδ1^+^ TCR repertoires but different from unfocussed cord blood Vδ1^+^ TCR repertoires (Fig. S2F).

**Fig. 3:**
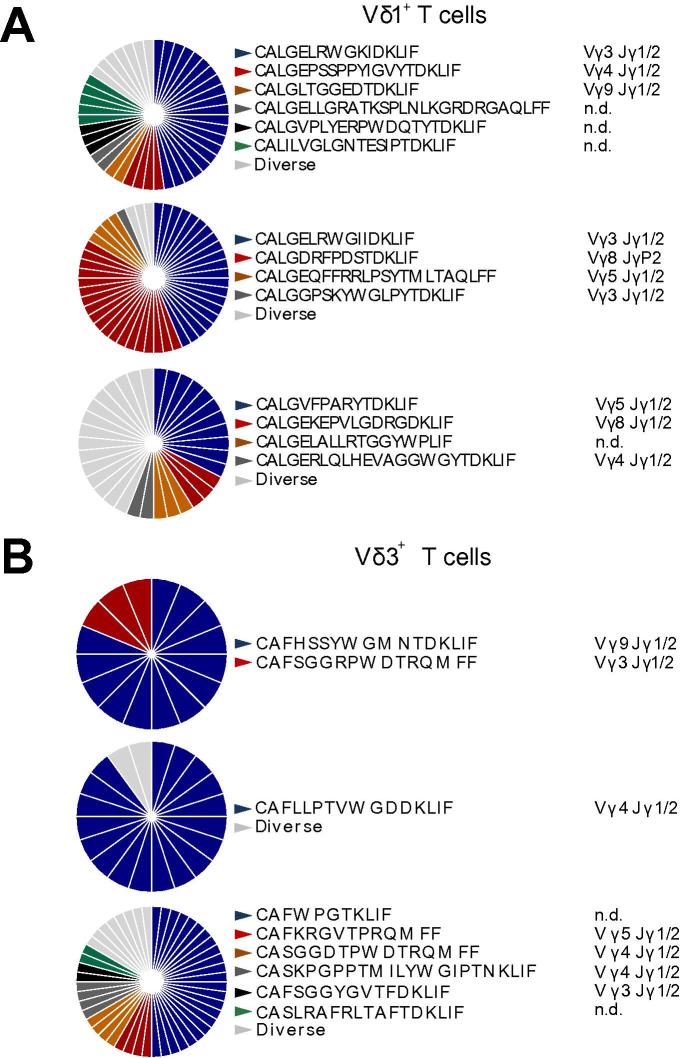
**Single-cell TCR sequencing reveals clonal focussing in Vδ2^−^ γδ T cells.** Clonal focussing of intrahepatic (A) Vδ1^+^ and (B) Vδ3^+^ cells determined by single-cell TCR sequencing analysis of CDR3δ. Each colour represents an individual CDR3δ, with clonal sequences labelled beside each chart (from 16–42 single cells per population, as indicated; with each pie chart representing an independent donor). CDR3, complementarity determining region 3; TCR, T cell receptor. (This figure appears in colour on the web.)

This distinctive clonal dominance was unequivocally confirmed by sorting single intrahepatic Vδ1^+^ and Vδ3^+^ T cells and performing single-cell TCR sequencing. This approach highlighted that intrahepatic Vδ1^+^ and Vδ3^+^ (Fig. 3) T cell populations were composed of a small number of dominant clonotypes, using a variety of functional Vγ and Jγ gene segments. We also confirmed that concurrent clonal focussing can occur in both Vδ1^+^ and Vδ3^+^ TCR repertoires in the same donors (Fig. S3A). Moreover, analysis of CDR3δ sequences revealed substantial complexity. As in peripheral blood, CDR3δ1 were long, frequently using two diversity (D) gene segments and containing extensive non-templated nucleotide (nt) additions (Table. S1). CDR3δ3 sequences were generally shorter than CDR3δ1 sequences and contained fewer non-templated nt (Table. S2; Fig. S3B), though there was no evidence of CDR3δ3 length restriction, in contrast to CDR3γ9 sequences in Vγ9^+^/Vδ2^+^ T cells.^16^ These data highlight the private nature of expanded clonotypes in intrahepatic Vδ2^−^ TCR repertoires and the broad range of Vγ chains that they collectively utilise.

**Fig. 4:**
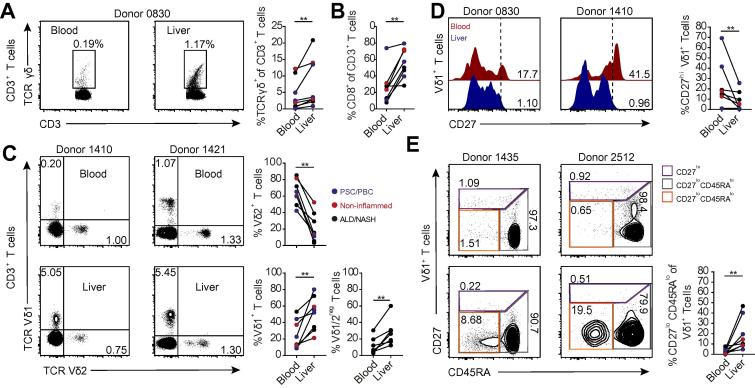
**Intrahepatic Vδ1^+^ T cells are phenotypically distinct from those in matched blood.** Representative flow cytometry plots and summary data of the enrichment of (A) γδ TCR^+^ and (B) CD8^+^ cells in donor matched liver and peripheral blood samples (n = 8). (C) Representative flow cytometry plots and summary data of the enrichment of Vδ1^+^ and Vδ1^−^Vδ2^−^ or contraction of Vδ2^+^ T cells in liver (lower plots) and donor matched peripheral blood (upper plots) (n = 8). (D) Representative histograms and summary data of the frequency of CD27^hi^ Vδ1^+^ T cells derived from donor matched liver and peripheral blood samples (n = 7). (E) Representative flow cytometry plots and summary data of CD27^lo/−^ CD45RA^hi^ and CD45RA^lo^ populations within donor matched liver (lower panels) and peripheral blood (upper panels) Vδ1^+^ T cells (n = 8). Data analysed by Mann-Whitney U test, **p <0.01. TCR, T cell receptor. (This figure appears in colour on the web.)

We next assessed the relationship between peripheral blood and intrahepatic Vδ1^+^ TCRs in the same individuals. Flow cytometry analysis of these matched samples indicated the enrichment of γδ T cells in the liver (Fig. 4A), which occurred alongside the previously noted enrichment of CD8^+^ αβ T cells (Fig. 4B).[25], [26], [27] Moreover, while Vδ1^+^ T cells were specifically enriched there was an overall reduction in the proportion of infiltrating Vδ2^+^ T cells in the liver compared to the blood (Fig. 4C). Peripheral blood Vδ1^+^ T cells comprise both clonotypically focussed effector and separate TCR-unfocussed naïve sub-compartments, which can be delineated based on distinct CD27^lo/−^ CD45RA^+^ and CD27^hi^ CD45RA^+/−^ expression patterns, respectively.^16^ We assessed liver and blood Vδ1^+^ T cells for the expression of CD27 and CD45RA surface markers (Fig. 4D-E); we noted a loss of CD27^hi^ Vδ1^+^ T cells (Fig. 4D) in intrahepatic γδ T cells, consistent with the lower diversity we observed in liver TCR repertoires than that of peripheral blood. While CD27^lo/−^ CD45RA^hi^ cells were present in both liver and blood, we noted the presence of an intrahepatic CD27^lo/−^ CD45RA^lo/−^ Vδ1^+^ T cell population that was present in all livers to varying degrees, but that was found at only very low levels in peripheral blood (Fig. 4E). The extent of this enrichment in liver was unaffected by liver disease aetiology (Fig. 4E) or CMV infection (Fig. S4).

**Fig. 5:**
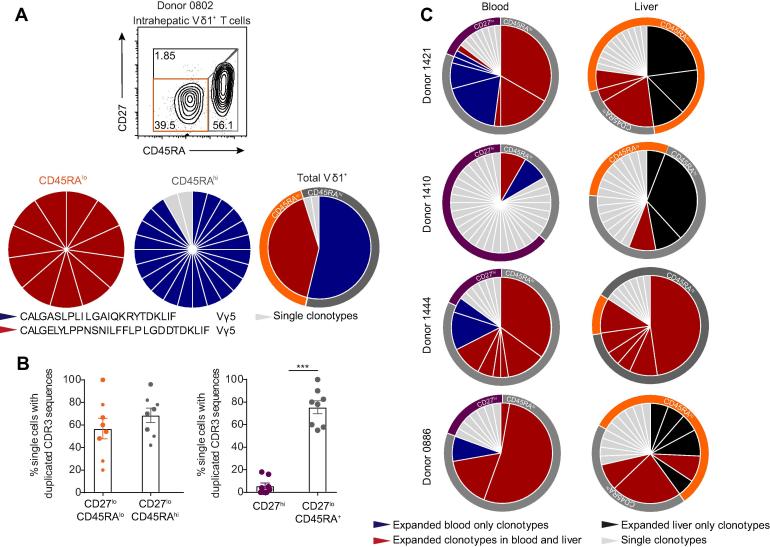
**Intrahepatic Vδ1 T cells contain clonotypes both distinct and overlapping with the blood.** (A) Clonal focussing of intrahepatic Vδ1^+^ CD27^lo/−^ CD45RA^lo^ (n = 11 single cells) and CD27^lo/−^ CD45RA^hi^ (n = 24 single cells) cells determined by single-cell TCR sequencing analysis of CDR3δ. Each colour represents an individual CDR3δ, with clonal amino acid sequences labelled below the chart. Total Vδ1^+^: TCR sequence data was combined with flow cytometry data to generate the two layered pie, linking clonotype (inner pie chart) to phenotype (outer pie chart). (B) Assessment of clonality by single-cell TCR sequencing analysis of CD27^lo/−^ CD45RA^lo^, CD27^lo^CD45RA^hi^ and CD27^hi^ Vδ1^+^ T cells sorted from liver and donor matched blood (n = 8). (C) Comparison of the relationship between phenotype (outer pie chart) and clonality (inner pie chart) determined by phenotype-linked indexed single-cell TCR sequencing analysis, in donor matched peripheral blood (upper) and liver (lower) Vδ1^+^ T cells, classified according to clone presence within liver and/or blood compartments. Error bars indicate mean ± SEM; data analysed by Mann-Whitney U test, ***p <0.001. CDR3, complementarity determining region 3; TCR, T cell receptor. (This figure appears in colour on the web.)

We then explored the clonality of intrahepatic CD27^lo/−^ CD45RA^hi^ and CD27^lo/−^ CD45RA^lo/−^ populations by single-cell TCR sequencing. In a representative liver sample, sorted intrahepatic CD27^lo/−^ CD45RA^lo^ and CD27^lo/−^ CD45RA^hi^ Vδ1^+^ T cell populations each comprised single prominent, distinct clonotypes using single-cell sort identities (*i.e.* CD45RA^hi^ or ^lo^), allowing the direct alignment of clonotype to phenotype at the single-cell level (Fig. 5A). Notably, within intrahepatic γδ T cells, both the CD45RA^hi^ and CD45RA^lo^ populations were predominantly clonally expanded (Fig. 5A; B, left panel). Consistent with previous findings,^16^ in blood the CD27^hi^ compartment (reduced in frequency in liver) was polyclonal, whereas the CD27^lo/−^ CD45RA^hi^ compartment was dominated by clonal expansions (Fig. 5B, right panel); notably the CD27^lo/−^ CD45RA^lo^ compartment was essentially absent in blood. We then systematically examined the relationship between clonotypic and phenotypic identity from matched pairs of blood and liver Vδ1^+^ γδ T cells (Fig. 5C). Overall in our paired samples, we identified clonotypes present in both the blood and liver, however importantly we also identified clonotypes unique to either liver or blood (Fig. 5C). The phenotype of clonotypes found only in the blood or shared between blood and liver generally mapped to the CD27^lo/−^ CD45RA^hi^ compartment found both in blood and liver. In contrast, the clonotypes present exclusively in the liver mapped between CD27^lo/−^ CD45RA^lo^ and CD27^lo/−^ CD45RA^hi^ compartments, with a trend towards a CD27^lo/−^ CD45RA^lo^ phenotype (Fig. 5C). As examples, the highly expanded Vδ1 CALGGGGFPQKPGGAGPPTAQLFF and CALGEHPHFFLHLIGTIKLIF clonotypes present in the livers of Donor 0886 and Donor 1421 (both ALD) respectively were CD27^lo/−^ CD45RA^hi^ in phenotype and also present in the respective matched peripheral blood samples, whereas in each case liver-restricted expanded clonotypes were also observed, but predominantly CD27^lo/−^ CD45RA^lo^ (Fig. S5A). Taken together, while considerable clonotypic overlap between liver and blood subsets is observed, we identified a distinct population of intrahepatic CD27^lo/−^ CD45RA^lo^ Vδ1^+^ T cells largely absent from the blood, and which frequently contains TCRs restricted to the liver. This paradigm is likely to extend to intrahepatic Vδ3^+^ γδ T cells, which also exhibited a significant proportion of CD45RA^lo^ cells (Fig. S5B).

**Fig. 6:**
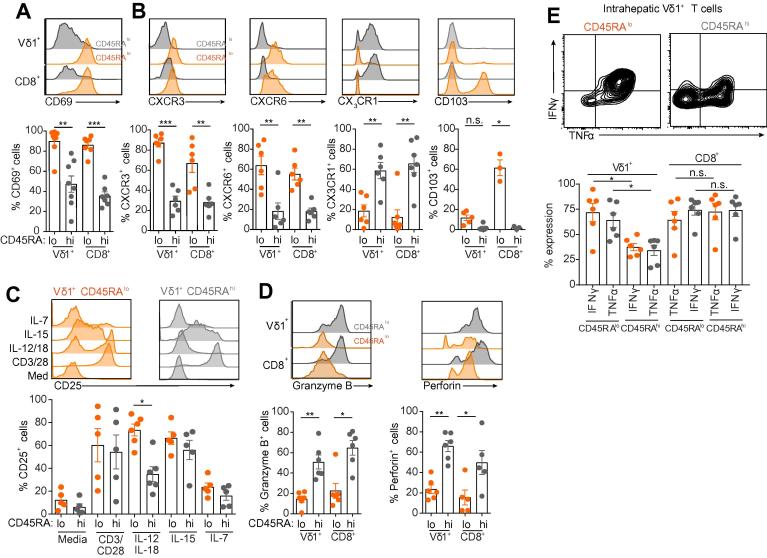
**Intrahepatic Vδ1^+^ T cells segregate into cytokine producing and cytotoxic subsets.** (A) Representative histograms from one donor and summary data of CD69 surface expression by CD45RA^lo^ (orange) and CD45RA^hi^ (grey) intrahepatic Vδ1^+^ and CD8^+^ T cells (n = 8). (B) As in (A), but displaying representative histograms and summary data for CXCR3, CXCR6 and CX_3_CR1 surface expression by intrahepatic Vδ1^+^ and CD8^+^ T cells (n = 6). (C) Representative histograms and summary data from sorted intrahepatic CD3^+^ T cells were incubated with indicated medium, cytokines or anti-CD3/CD28 beads for 72 h. CD45RA^lo^ (orange) and CD45RA^hi^ (grey) Vδ1^+^ T cells were then assessed for the upregulation of the T cell activation marker CD25 (n = 5–6). (D) Representative histograms and summary data for intracellular granzyme B and perforin expression by CD45RA^lo^ (orange) and CD45RA^hi^ (grey) intrahepatic Vδ1^+^ and CD8^+^ T cells (n = 5–6). (E) Representative flow cytometry plot and summary data of intrahepatic CD3^+^ T cells stimulated with PMA/Ionomycin and assessed for the production of intracellular IFNγ and TNFα in CD45RA^lo^ (orange) and CD45RA^hi^ (grey) Vδ1^+^ and CD8^+^ cells (n = 6). Error bars indicate mean ± SEM; data analysed by Kruskal-Wallis ANOVA with Dunn’s post-test comparisons, n.s. *p* >0.05, *p <0.05, **p <0.01 and ***p <0.001. (A–E) Disease aetiologies analysed included ALD, NASH, PSC, and normal liver no significant differences were observed between different individual disease groups in any of the comparisons highlighted. ALD, alcoholic liver disease; IFN, interferon; NASH, non-alcoholic steatohepatitis; PSC, primary sclerosing cholangitis; TNFα, tumour necrosis factor alpha. (This figure appears in colour on the web.)

We sought to further characterise intrahepatic CD27^lo/−^ CD45RA^lo^ and CD27^lo/−^ CD45RA^hi^ Vδ1^+^ T cells for markers associated with tissue retention. Firstly, while the surrogate marker of tissue-resident memory T cells (T_RM_), CD69, was expressed widely by Vδ1^+^ T cells, it was markedly higher on CD27^lo/−^ CD45RA^lo^ Vδ1^+^ T cells and comparable to CD45RA^lo^ CD8^+^ αβ T cells (Fig. 6A). In keeping with functional tissue retention, Vδ1^+^ T cells expressed CXCR3 and CXCR6, with expression predominantly associated with the CD27^lo/−^ CD45RA^lo^ population (Fig. 6B). In contrast, the endothelial homing receptor CX_3_CR1 (highly expressed by peripheral blood CD27^lo/−^ CD45RA^hi^ Vδ1^+^ T cells^16^) was retained on intrahepatic CD27^lo/−^ CD45RA^hi^ cells but was markedly reduced on CD27^lo/−^ CD45RA^lo^ Vδ1^+^ T cells (Fig. 6B). Interestingly, intrahepatic CD45RA^lo^ Vδ1^+^ T cells did not express significantly more CD103 than CD45RA^hi^ Vδ1^+^ T cells, which contrasts with CD8^+^ CD45RA^lo^ T cells isolated from the same livers (Fig. 6B). We next assessed the functionality of intrahepatic Vδ1^+^ T cell populations by *ex vivo* stimulation with recombinant cytokines or by TCR activation. Following TCR stimulation, intrahepatic Vδ1^+^ T cell populations in general strongly upregulated the T cell activation marker CD25, with equivalent responses in CD8^+^ αβ T cells from the same samples, although Vδ1^+^ T cells from some liver samples responded more robustly than others. Importantly, intrahepatic CD27^lo/−^ CD45RA^lo^ Vδ1^+^ T cells displayed a greater sensitivity to innate associated cytokines IL-12 and IL-18, than CD27^lo/−^ CD45RA^hi^ Vδ1^+^ T cells (Fig. 6C). Notably, peripheral blood CD27^lo/−^ CD45RA^hi^ Vδ1^+^ T cells are unresponsive to IL12/IL-18 stimulation.^16^ In keeping with a clonally expanded intrahepatic Vδ1^+^ T cell population, significant responses were observed with IL-15 but not IL-7 cytokines (Fig. 6C). We next assessed effector potential, by analysing intracellular expression of cytolytic granzyme B and perforin. Intrahepatic CD27^lo/−^ CD45RA^hi^ Vδ1^+^ T cells expressed marked levels of both effector molecules while CD27^lo/−^ CD45RA^lo^ Vδ1^+^ T cells had much lower expression (Fig. 6D). Conversely, stimulation of the CD27^lo/−^ CD45RA^lo^ population with PMA and ionomycin produced significantly more of the pro-inflammatory cytokines IFN-γ and TNFα than the CD27^lo/−^ CD45RA^hi^ population (Fig. 6E). These data suggest that intrahepatic CD27^lo/−^ CD45RA^lo^ Vδ1^+^ T cells have a more prominent tissue-associated phenotype than that of the CD27^lo/−^ CD45RA^hi^ Vδ1^+^ T cell population, which are more similar to peripheral blood CD27^lo/−^ CD45RA^hi^ Vδ1^+^ T cells. Moreover, these two populations possess either enhanced cytolytic (CD45RA^hi^) or pro-inflammatory cytokine (CD45RA^lo^) responses, suggesting distinct roles in intrahepatic immunity.

## Discussion

Tissue-associated T cells are thought to play a critical role in tissue immunosurveillance and homeostasis.[28], [29], [30] In mice, γδ T cells have been implicated in epithelial homeostasis,^31^ cutaneous wound healing^32^ and maintenance of gut mucosa,^33^ and have been highlighted as innate-like, expressing canonical TCRs.^34^ In humans, solid tissues are known to be enriched for γδ T cells but the immunobiology of the T cells present has remained largely unclear. Recent studies on Vδ1^+^ T cells, the canonical tissue-associated human γδ T cell subset, have revealed an adaptive biology.[16], [17] However, these results were based exclusively on peripheral blood Vδ1^+^ cells, and the immunobiology of solid tissue-associated Vδ1^+^ lymphocytes, often assumed to be innate-like, is of particular interest. We chose to probe these issues by characterising intrahepatic γδ T cells as a human model system.

We used NGS approaches to show the hepatic Vδ2^−^ compartment is comprised of highly clonal, private expansions, based on complex TCR rearrangements. Importantly these were evident in both diseased and healthy livers, with no skewing of the TCR repertoire chain usage observed between the two scenarios. Moreover, the proportion of Vδ2^−^ γδ T cells decreased upon liver inflammation compared with healthy livers, because of an influx of αβ T cells. Therefore, the accumulation of γδ T cells in human liver is not driven by the diseased hepatic microenvironment present in these patients, and may reflect a response to other immune challenges such as infection. Of relevance, CMV infection has recently been highlighted as one of a number of drivers of Vδ2^−^ T cell clonal expansion (specifically of Vδ1^+^ T cells) in peripheral blood.[16], [17] Moreover, studies on murine CMV have highlighted the potential of expanded γδ T cell subsets to populate a range of peripheral tissues, including the liver.[35], [36] These observations raise the significant possibility that the expanded clonotypes that contribute so dominantly to human intrahepatic γδ T cells both in normal and diseased settings have arisen due to previous infections. Consistent with this, Vδ1^+^ γδ T cells were significantly enriched in liver explants from CMV^+^ *vs.* CMV^−^ donors. Therefore, CMV represents one likely driver of Vδ1^+^ infiltration in the liver. However, it is notable that similar clonotypic focussing and immunophenotypic profiles of intrahepatic Vδ2^−^ T cells were observed in both CMV^+^ and CMV^−^ individuals, consistent with the idea that the Vδ2^−^ subset can mount tissue-localised responses to multiple infections. This mirrors the situation with human Vδ1^+^ T cells in peripheral blood, where although CMV is linked with an increased proportion of Vδ1^+^ T cells[16], [37] and clearly drives clonal expansions of Vδ1^+^ clonotypes,^17^ such expansions are commonly observed in CMV^−^ individuals, suggestive of other infectious drivers.^16^ While the candidate drivers of intrahepatic Vδ2^+^ T cell expansion would include HCV/HBV, notably we did not study HCV/HBV-related liver disease, and therefore other non-CMV/HCV/HBV drivers must exist. In principle, an alternative to infection representing a main driver of Vδ2^−^ clonal expansion is that intrahepatic Vδ2^−^ T cells are populated in the liver during development. However, both their Vδ2^−^ chain usage and the highly complex nature of the intrahepatic Vδ2^−^ TCR CDR3 regions would argue against this possibility, since foetal γδ TCRs would be expected to utilise more simple CDR3 sequences and have also been highlighted as predominantly Vδ2^+^,^38^ thereby highlighting post-natal stimuli such as infection as a more likely underlying driver.

Given previous observations regarding peripheral blood Vδ1^+^ T cells,^16^ which like those in the liver were frequently highly clonal and also featured private expansions based on complex TCR rearrangements, a key question was the extent to which liver Vδ2^−^ γδ T cells mirrored those in the blood. Our study provides compelling evidence that despite the profound link between the liver and the peripheral circulatory system, there is a distinct profile of Vδ2^−^ γδ T cells in each compartment, indicative of compartmentalisation of certain Vδ2^−^ subsets.

Comparison of matched liver and blood samples indicated the differentiation status of the Vδ2^−^ T cell subset was distinct in each compartment. Strikingly, liver Vδ2^−^ T cells were uniformly CD27^lo/−^, a phenotype previously linked to a clonally expanded effector subset present in peripheral blood, and essentially entirely lacked the CD27^hi^ subset, even when such populations were relatively prevalent in matched blood. Previously we have shown that CD27^hi^ Vδ1^+^ T cells in peripheral blood are TCR-diverse and naïve in phenotype. Consistent with selective exclusion of this clonally diverse CD27^hi^ naïve population, liver Vδ2^−^ cells lacked CCR7, CD62L and CD27 present on such naïve populations, and diversity metrics indicated liver Vδ2^−^ T cells displayed an even more focussed repertoire in liver than in peripheral blood. Furthermore, the phenotype of liver Vδ2^−^ T cells closely matched that of peripheral blood CD27^lo/−^ Vδ1^+^ T cells, and there was substantial clonotypic overlap between these two populations. While we cannot exclude the possibility that such hepatic CD27^lo/−^ originated in the liver, these results support the concept that at least some hepatic CD27^lo/−^ cells may derive from those present in peripheral blood. Such a scenario would fit an adaptive model whereby naïve peripheral blood Vδ2^−^ CD27^hi^ cells, which express secondary lymphoid homing markers but are devoid of CX_3_CR1, recirculate between blood and lymph, whereas the peripheral blood CD27^lo/−^ population, which is clonally expanded and likely antigen-experienced, is capable of accessing solid tissues, potentially because of increased CX_3_CR1 expression, and may also upregulate tissue retention markers following liver localisation.

A second indication of compartmentalisation was that in addition to being devoid of CD27^hi^ naïve cells, the hepatic Vδ2^−^ T cell compartment comprised both a CD45RA^hi^ and also a distinct CD45RA^lo^ subset. By contrast, the peripheral blood CD27^lo/−^ Vδ1^+^ cells are almost entirely CD45RA^hi^. Importantly, CD45RA^hi^ clonotypes overlapped substantially between blood and liver within individuals. Such cells in the peripheral blood express a high level of the endothelial homing receptor CX_3_CR1 as well as increased CD16, low CD27/28, low CD127, and enhanced levels of adhesion molecules relative to naïve CD27^hi^ cells.^16^ While this could suggest capability of homing from peripheral blood to tissues, alternatively it could imply a vascular association, as has been suggested for effector memory CD8 T cells,^39^ which include virus-specific CD8^+^^40^ and CD4^+^^41^ T cell subsets. The predominantly sinusoidal localisation of these cells identified in this study is consistent with this possibility, and may suggest a role in immunosurveillance at this site, as suggested for NKTs.^42^ In light of the recent report that Vδ1^+^ clonotypes can expand in response to CMV,^17^ a virus that infects the endothelial compartment *in vivo*, and our observation here that Vδ1^+^ T cells are enriched in CMV^+^ *vs.* CMV^−^ liver explants, these findings suggest this subset may contribute to unconventional T cell protection of the vascular niche, including within solid tissues, against chronic viral infection. Moreover, the observation CMV serostatus correlates with an enhanced proportion of intrahepatic Vδ1^+^ T cells but not with a disturbed CD45RA^hi^ *vs.* CD45RA^lo^ Vδ1^+^ ratio might suggest the potential within both phenotypic sub-compartments to respond to CMV.

In contrast to CD45RA^hi^ clonotypes and consistent with a reduced frequency of CD45RA^hi^ Vδ2^−^ cells in liver compared to peripheral blood, the same analyses of matched blood/liver samples revealed CD45^lo^ clonotypes were enriched for those restricted to the liver. In addition, this liver CD45RA^lo^ compartment frequently contained clonal expansions. These cells demonstrate striking phenotypic correlation with liver-resident lymphocytes identified in previous studies, including enhanced expression of CD69, CXCR3 and CXCR6, which has been noted in liver-resident NK populations[43], [44] and CD8^+^ αβ populations.^25^ CD27^lo/−^ CD45RA^lo^ Vδ2^−^ T cells may therefore represent a liver-resident subset, although conceivably they may be able to access other solid tissues. Of note, CD45RA^lo^ Vδ1^+^ T cells exhibited considerably lower expression of CD103 relative to their CD8^+^ counterparts, suggesting other mechanisms may underly their tissue retention. The origin of this subset is unclear. One possibility is that it originates from a subset of blood CD45RA^+^ cells that alter phenotype once in tissues and are retained there, perhaps following activation in the hepatic microenvironment. This route of generation is supported by our detection of liver-restricted clonotypes in both the CD45RA^lo^ and CD45RA^hi^ compartments. In addition, it is possible they may be locally generated. Moreover, recent reports highlight that a liver-resident phenotype can be induced in CD8^+^ αβ T cells via IL-15 followed by TGF-β signalling,^25^ and based on the parallels between Vδ1^+^ and CD8^+^ αβ T cells identified in this study, a similar mechanism may be at work here.

Our results also highlight that hepatic γδ T cells are functionally distinct from equivalent subsets in peripheral blood. While still responsive to TCR stimulation/co-stimulation, compared to blood Vδ2^−^ T cells they displayed markedly increased responsiveness to IL-12/IL-18 in line with CD8^+^ T cells isolated from the same tissue. This responsiveness extended to the liver-restricted CD45RA^lo^ subset, which appeared to display enhanced production of pro-inflammatory cytokines relative to CD45RA^hi^ cells. These observations suggest CD45RA^hi^ and CD45RA^lo^ subsets may have different roles, the former more vascular focussed and cytotoxic, the latter an immunoregulatory tissue-associated subset more focussed on cytokine production and potential induction of a wider T cell response to stress challenges. It is unclear if these distinct features stem directly from the nature of the clonotypes present and their antigenic targets, or whether they reflect the influence of hepatic microenvironmental factors that may also influence intrahepatic retention.^45^

Importantly, we note several limitations of our study. Firstly, all diseased samples were derived from end-stage liver disease. While the closely matched clonotypic focussing and immunophenotypic profiles present in normal tissue would predict similar profiles at earlier disease stages, we cannot exclude the possibility that disease stage influences the nature of the intrahepatic γδ T cell population, and use of biopsy material from early disease stages with longitudinal follow-up could be an interesting avenue of future investigation. Secondly, while we examined several disease pathologies, these were predominantly restricted to fatty/alcoholic liver disease (ALD, NAFLD) or autoimmune liver disease (AIH, PBC, or PSC). While HCV/HBV+ liver samples showed similar frequencies of γδ T cells, we did not study γδ T cell immunophenotype or clonotypic focussing in such samples and cannot therefore exclude the possibility that HCV/HBV infection may drive development of distinct intrahepatic γδ T cell profiles^47^ or clonality, although we hypothesise they would follow broadly similar principles to those observed in this study; moreover, while we did not observe differences between the different disease types we did analyse, conceivably with larger samples sizes differences may have emerged, for example in the extent of γδ TCR clonotypic focussing or γδ T cell phenotypes. Finally, a comparison of the data presented here with γδ T cell clonotype and immunophenotype profiles in other solid tissues, including during chronic inflammation, would shed light on tissue-specific γδ T cell responses.

Our study establishes that in humans, clonally expanded γδ T cell effector subsets can be selectively deployed to at least some solid tissues, including the liver, thereby providing ongoing immune surveillance against previously encountered infectious or non-infectious challenges, with CMV infection one likely driver of Vδ1^+^ T cell intrahepatic infiltration. Importantly, both Vδ1^+^ and Vδ3^+^ intrahepatic T cell compartments displayed clonotypic expansion and a CD45RA^lo^ subset, suggesting their immunobiology may be closely aligned. Moreover, the finding that intrahepatic γδ T cell subsets can be phenotypically, clonotypically and functionally distinct from those in peripheral blood suggests distinct contributions to intrahepatic immune responses, and provides a basis for future investigation of human tissue-resident γδ T cell populations. Notably, γδ T cells are of increasing therapeutic interest, due partly to their potential to mount either anti-tumour,[47], [48], [49] or alternatively immunosuppressive^50^ responses, but also their MHC-unrestricted recognition of target cells, which raises the prospect of broad applicability of γδ T cell-based therapies in patient cohorts. Our finding that there appears to be selective recruitment of γδ T cell subsets of an effector phenotype into the hepatic pool may inform design of γδ T cellular therapies that rely on administration/expansion of systemic γδ T cells. Secondly, the finding that a number of distinct differentiation states exist within the Vδ1^+^ compartment (including naïve, circulating effector, tissue-resident effector) indicates a degree of plasticity that could be investigated further and potentially exploited therapeutically, either to increase immunosuppressive functionality during inflammatory liver disease, or for improved anti-tumour effector function in liver cancer. Finally, our finding that CMV infection represents one likely factor driving infiltration of potentially highly inflammatory Vδ1^+^ T cells into the liver could have clinical relevance in chronic liver disease and CMV-associated hepatitis. Specifically, future studies correlating CMV titres with biomarkers of liver damage and with Vδ1^+^ γδ T cell frequency may shed light on whether the γδ T cell response to CMV infection impacts the severity of chronic liver disease.

## Financial support

The work was supported by a Medical Research Council funded PhD studentship to S.H., by Wellcome Trust Investigator award funding to B.W., supporting M.D. and C.W. (Grant code: 099266/Z/12/Z), by a Russian Foundation for Basic Research grant 17–04–01994 (S.K.) and 17-54-10018 (D.C.), by a Medical Research Council Clinician Scientist award to Y.H.O (G1002552), and by the Ministry of Education, Youth and Sports of the Czech Republic under the project CEITEC 2020, LQ1601 (D.C.).

## Conflict of interest

The authors declare no conflicts of interest that pertain to this work.

Please refer to the accompanying ICMJE disclosure forms for further details.

## Authors’ contributions

Y.O. and B.W. supervised the project; S.H., M.D., C.W., B.W. and Y.O. designed experiments; S.H. performed experiments, prepared liver tissue samples and analysed data; C.W. and M.D. performed experiments and analysed data; H.J. provided liver samples and technical assistance; S.K. and D.C. analysed and interpreted TCR deep sequencing data; S.H., C.W. and M.D. wrote the draft and prepared figures; M.D., C.W. S.H, Y.O and B.W. wrote the final manuscript; and all authors provided critical review of the manuscript.
