# Supplementary material for: Human liver infiltrating γδ T cells are composed of clonally expanded circulating and tissue-resident populations
Source: J Hepatol. 2018 Sep;69(3):654–65. doi: 10.1016/j.jhep.2018.05.007 (PMC6089840; doi:10.1016/j.jhep.2018.05.007)
Supplement: Supplementary data 2 [file CTAT_table.pdf]

Journal of Hepatology

CTAT methods

Tables for a "Complete, Transparent, Accurate and Timely account" (CTAT) are now mandatory for all revised submissions. The aim is to enhance the reproducibility of methods.

- Only include the parts relevant to your study
- Refer to the CTAT in the main text as 'Supplementary CTAT Table'
- Do not add subheadings
- Add as many rows as needed to include all information
- Only include one item per row

If the CTAT form is not relevant to your study, please outline the reasons why:

All methods are adequately described in the manuscript and therefore we have not included reference to this CTAT form. This includes the TCR sequence data deposition codes, which are referred to in the 'Data Availability' section of the Material and Methods section.

1.1 Antibodies

Name	Citation	Supplier	Cat no.	Clone no.
A number of antibodies were used, as specified in the manuscript.				

1.2 Cell lines

Name	Citation	Supplier	Cat no.	Passage no.	Authentication test method
As specified in manuscript.					

1.3 Organisms

Name	Citation	Supplier	Strain	Sex	Age	Overall n number
NA						

1.4 Sequence based reagents

Name	Sequence	Supplier
As specified in manuscript.		

1.5 Biological samples

Description	Source	Identifier

Samples included human liver, predominantly from end-stage liver disease explants, as stated in the manuscript.		

1.6 Deposited data

Name of repository	Identifier	Link
NIH NCBI sequence read archive (SRA). The sequence data that support the findings of this study have been deposited in the NIH NCBI sequence read archive (SRA) database with the primary accession code SRP113556 and SRP096009, for γδ TCR repertoires	SRP113556 and SRP096009	**https://www.ncbi.nlm.nih.gov/sra**

1.7 Software

Software name	Manufacturer	Version
NA		

1.8 Other (e.g. drugs, proteins, vectors etc.)

NA		

1.9 Please provide the details of the corresponding methods author for the manuscript:

Stuart Hunter

2.0 Please confirm for randomised controlled trials all versions of the clinical protocol are included in the submission. These will be published online as supplementary information.

NA
