# Supplementary material for: Human liver infiltrating γδ T cells are composed of clonally expanded circulating and tissue-resident populations
Source: J Hepatol. 2018 Sep;69(3):654–65. doi: 10.1016/j.jhep.2018.05.007 (PMC6089840; doi:10.1016/j.jhep.2018.05.007)
Supplement: Supplementary Figs. S1–S5 and Tables S1–S2 [file mmc1.pdf]

Human liver infiltrating γδ T cells are composed of clonally expanded circulating and tissue-resident populations

Stuart Hunter, Carrie R. Willcox, Martin S. Davey, Sofya A. Kasatskaya, Hannah C. Jeffery, Dmitriy M. Chudakov, Ye H. Oo, Benjamin E. Willcox

Table of contents

Fig. S1……………………………………………………………………………………2

Fig. S2……………………………………………………………………………………3

Fig. S3……………………………………………………………………………………4

Fig. S4……………………………………………………………………………………5

Fig. S5……………………………………………………………………………………6

Table S1…………………………………………………………………………………7

Table S2…………………………………………………………………………………8

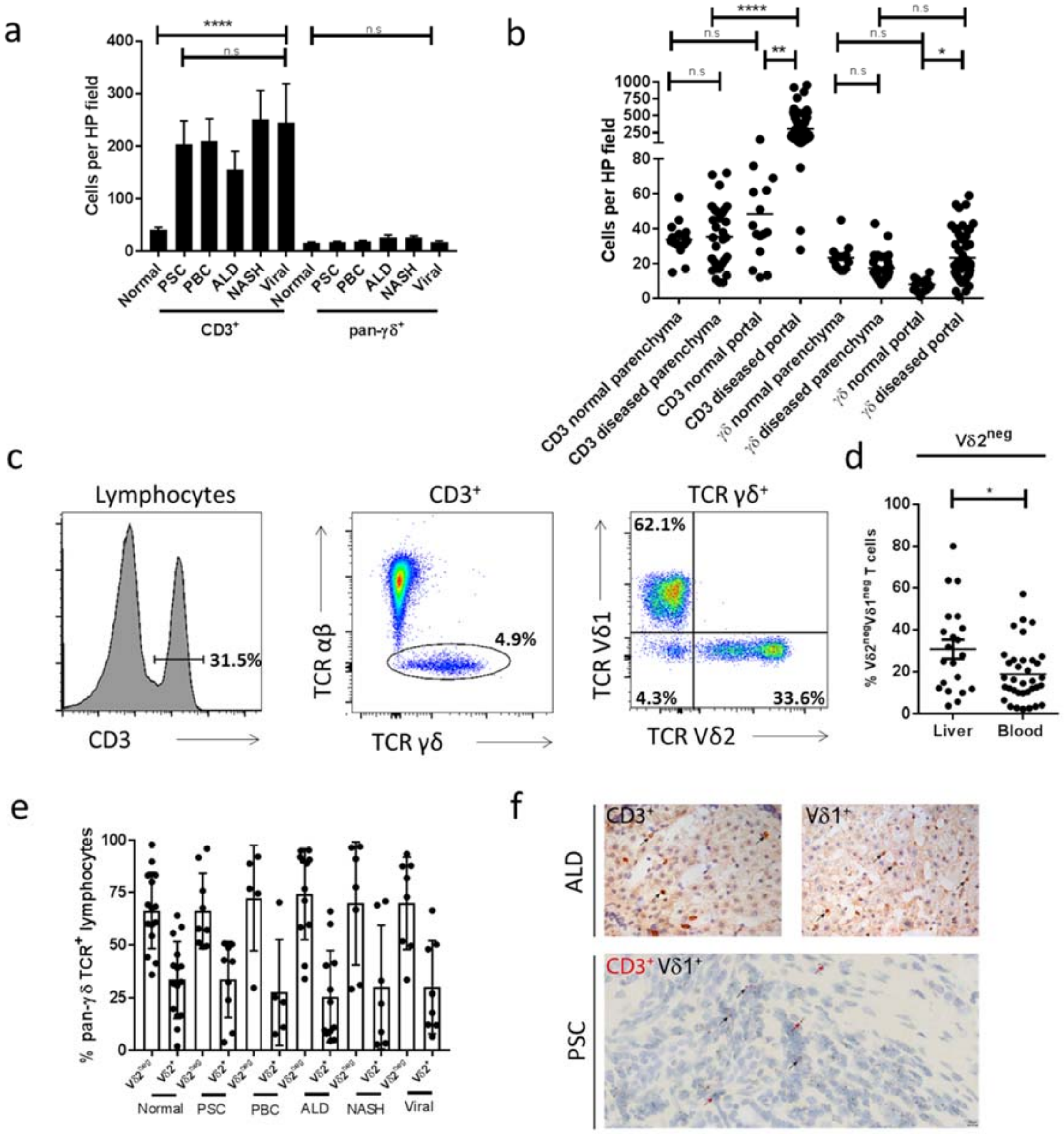

Fig. S1. Intrahepatic Vδ2^{neg} $\gamma\delta$ T cell proportions in diseased liver tissue.
(a) Densities of CD3^{+} and $\gamma\delta$ TCR^{+} cells in normal (n=21) PSC (n=13), PBC (n=13, ALD (n=12), NASH (n=12) and viral hepatitis (n=12) liver tissue. (b) Densities of CD3^{+} and $\gamma\delta$ TCR^{+} cells in parenchymal and portal areas from normal (n=15) and diseased (n=30) liver tissue. (c) Representative flow cytometry plots showing gating strategy used to identify intrahepatic CD3^{+} lymphocytes $\Rightarrow$ $\gamma\delta$ TCR^{+} $\alpha\beta$ TCR^{neg} $\Rightarrow$ Vδ1^{+}, Vδ2^{+} and Vδ1^{neg} Vδ2^{neg} T cells. (d) Comparison of Vδ2^{neg} Vδ1^{neg} $\gamma\delta$ T cells in explanted liver (n=21) and peripheral blood (n=35). (e) Comparison of Vδ2^{+} and Vδ2^{neg} proportions in $\gamma\delta$ TCR^{+} cells identified by flow cytometry from normal (n=15), PSC (n=9), PBC (n=5), ALD (n=12), NASH (n=7) and viral hepatitis (n=8) liver cell suspensions. (f) Representative staining for CD3^{+} (upper left) and Vδ1 TCR^{+} (upper right) cells on sequential FFPE sections from ALD liver tissue viewed at 20× magnification, and CD3 (brown) and Vδ1 TCR (red) transcripts by in-situ hybridisation of PSC tissue viewed at 40x magnification. Error bars indicate mean ± SEM; data analysed by Kruskal–Wallis ANOVA with Dunn's post-test comparisons (a-c) and Student's T test (e), n.s. $p>0.05$, *$p<0.05$, **$p<0.01$ and****$p<0.0001$.

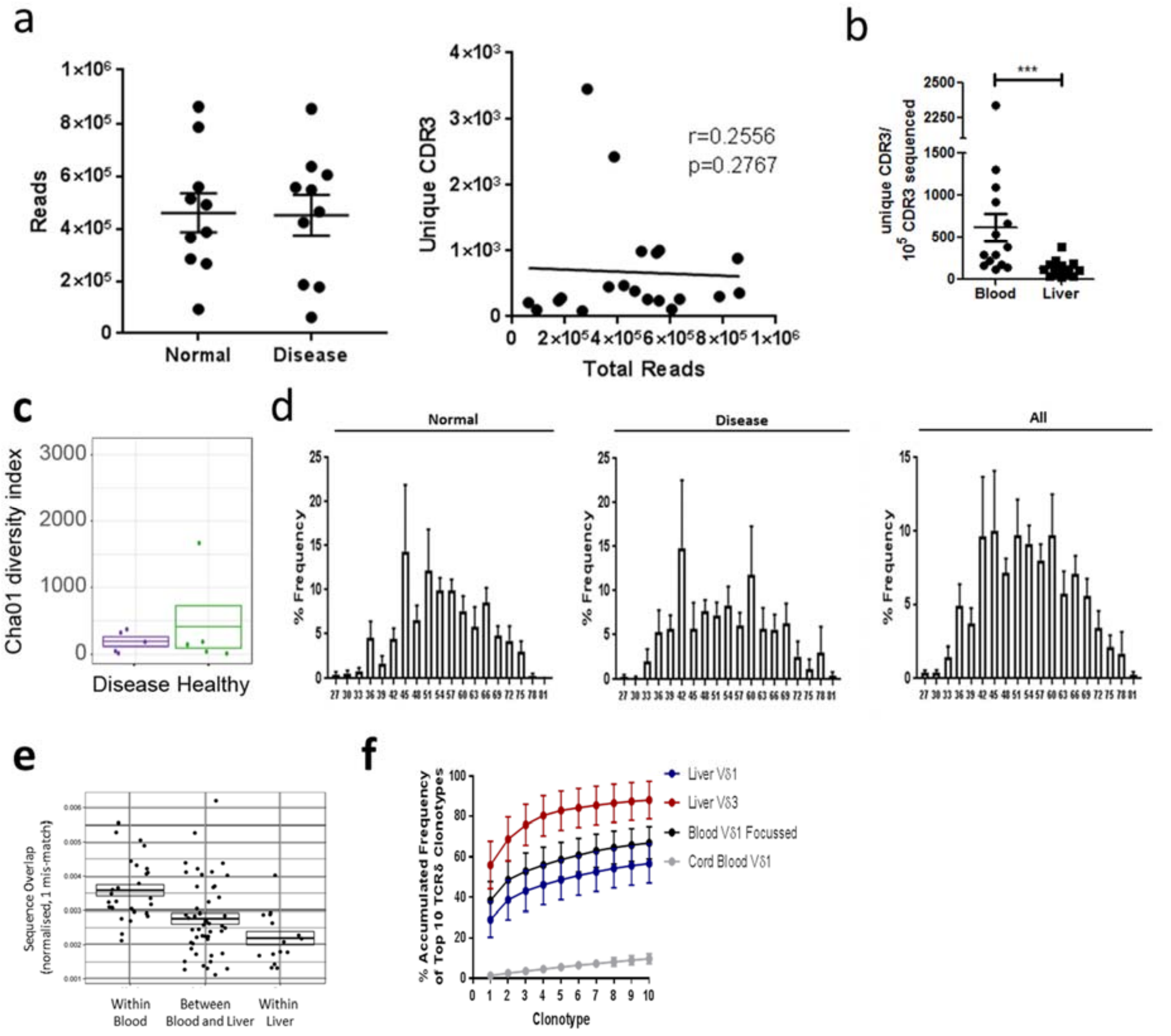

Fig. S2. TCR repertoire sequence analysis of intrahepatic Vδ2^{neg} γδ T cells.
(a) Comparison of combined CDR3δ and CDR3γ sequencing reads from Vδ2^{neg} T cells isolated from normal (n=5) and diseased (n=5) livers (left) and total sequencing reads against the number of unique CDR3s determined for each donor (n=10). (b) Comparison of the number of unique CDR3δ and CDR3γ sequences determined per 10^5 sequences from blood (n=7) and liver (n=10) derived Vδ2^{neg} T cells. (c) Comparison of Chao1 total Vδ2^{neg} T cell repertoire richness estimation (diversity) from diseased and normal livers. (d) Non-normalised length spectratyping for CDR3δ in normal (left, n=5), disease (centre, n=5) and combined (right, n=10) intrahepatic Vδ2^{neg} T cells. (e) Comparison of the proportion of sequences in the TCRγ repertoire that are observed in more than one donor (allowing up to one amino acid mismatch) within blood samples (n=7), liver samples (n=10), or in between liver and blood samples. (f) Accumulated frequencies occupied by the 10 most prevalent TCR Vδ1$^+$ and Vδ3$^+$ clonotypes in liver (n=10) and Vδ1$^+$ "focussed" blood (n=13) and cord blood (n=5) donors (both from Davey *et al*, 2017). Error bars indicate mean ± SEM; data analysed by spearman correlation and student's T test, ***$p<0.001$.

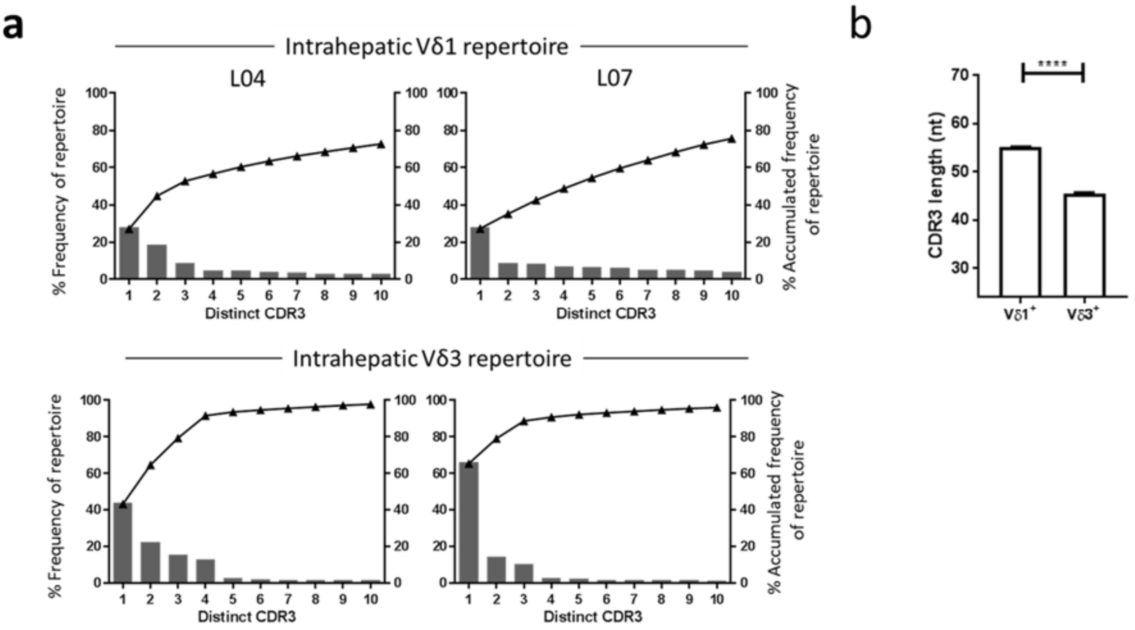

Fig. S3. Concurrent clonal focussing in Vδ2^{neg} γδ T cell subsets.
(a) Individual clone frequency (left y axis) and accumulated frequency (right y axis) for the 10 most prevalent TCR Vδ1^{+} (upper) and Vδ3^{+} (lower) clonotypes from two liver donors with appreciable Vδ3^{+} T cell populations. (h) Comparison of CDR3 lengths for Vδ1^{+} and Vδ3^{+} TCRs in liver samples (n=10). Error bars indicate mean ± SEM; data analysed by student's T test, ****$p<0.0001$.

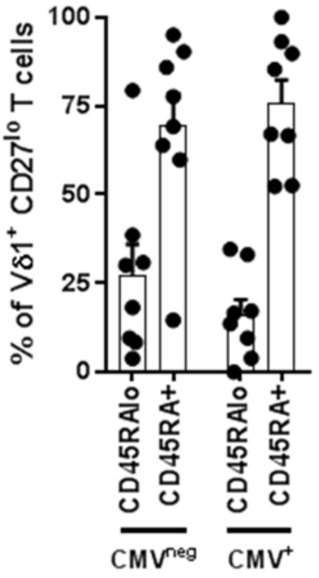

Fig. S4. CMV infection does not influence proportion of CD45RA^{lo} Vδ1^{+} γδ T cells.
Comparison of intrahepatic CD27^{lo} Vδ1^{+} T cell expression of CD45RA according to CMV status of liver donor (n=8 CMV^{neg} & CMV^{+}).

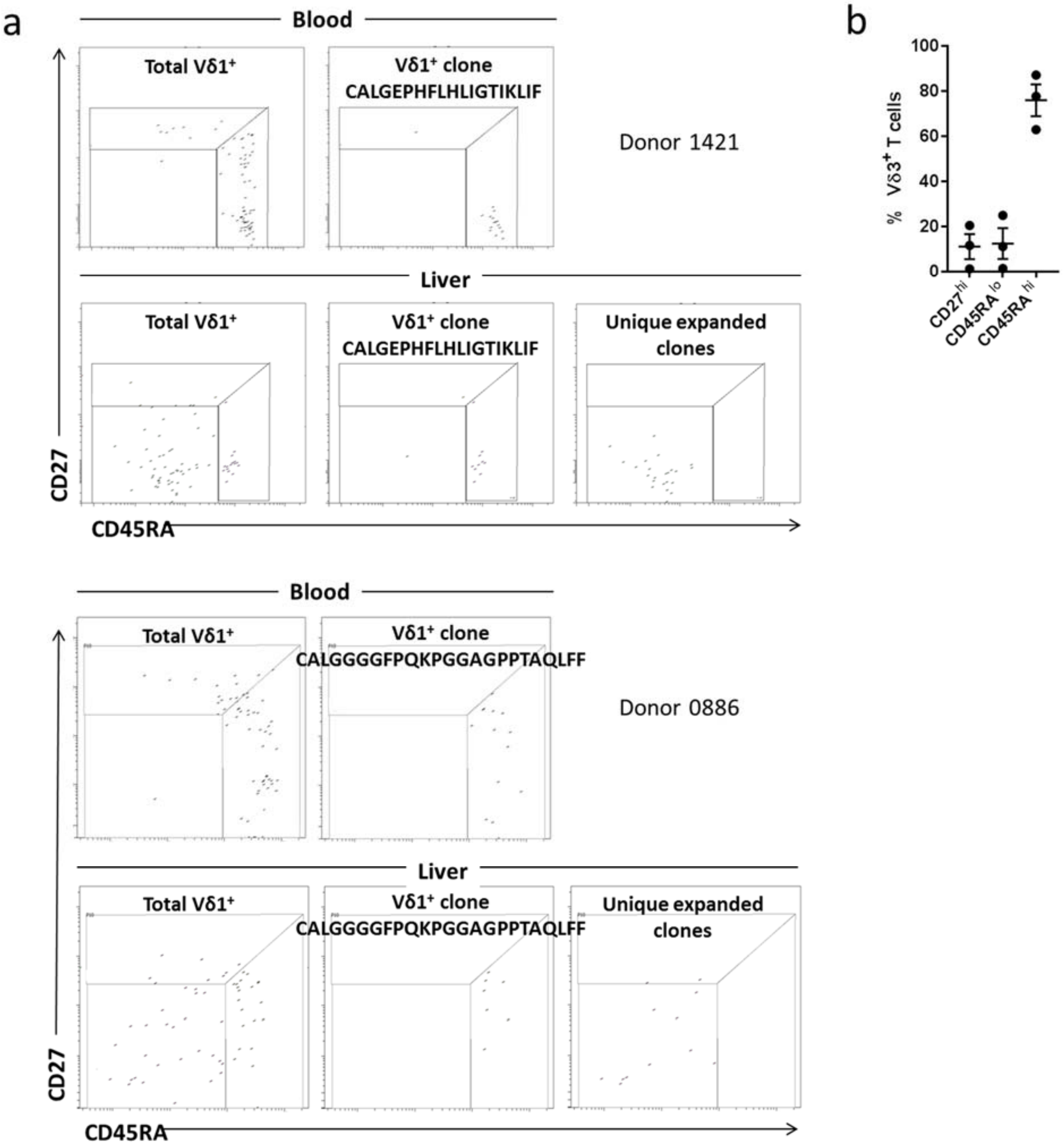

Fig. S5. Phenotype-linked indexed single cell TCR sequencing analysis.

(a) Representative flow cytometry plots from two donors showing expression of CD27 and CD45RA by single Vδ1$^+$ T cells sorted from donor matched blood and liver samples. Single cells were subsequently analysed by TCR sequencing and plots are shown for all cells (left), cells with most prevalent CDR3 sequence found in both liver and blood samples (centre) and cells identified only liver samples that displayed CDR3 sequences with at least one other cell (right). (b) Summary data of intrahepatic Vδ3$^+$ $\gamma\delta$ T cells phenotype according to expression of CD27 and CD45RA (n=3).

germline		TRDV					N/P	TRDD1	TRDD2	N/P	TRDD3	N/P			TRDJ										
	TRDV1	GCT	CTT	GGG	GAA	CT		GAAATAGT	CCTTCCTAC		ACTGGGGGATACG					AC	ACC	GAT	AAA	CTC	ATC	TRJD1			
															CT	TTG	ACA	GCA	CAA	CTC	TTC	TRJD2			
													C	TCC	TGG	GAC	ACC	CGA	CAG	ATG	TTT	TRJD3			
																							CDR3	N/P nt	
donor																							length	added	protein sequence
L01	TRDV1	GCT	CTT	GGG	GAA	C	GTCAGGCGTTAGGTA		CTTCC	CTCCGAA	ACTGGGGGATA	GTAAAAAT					C	GAT	AAA	CTC	ATC	TRJD1	72	30	CALGERQALGTSLRNWGIVKIDKLIF
L02	TRDV1	GCT	CTT	GGG	GAA	C	GTTC		CCTTCCT	GCCAAACGATATGGGGTGCT	GGGGATA	AGCCTCCCTT				AC	ACC	GAT	AAA	CTC	ATC	TRJD1	78	34	CALGERSLPAKRYGVLGISLPYTDKLIF
L03	TRDV1	GCT	CTT	GGG	GAA	C	ATAT		CTTCCTAC	GT	CTGG	ATCCGGCGCTAGA			T	TTG	ACA	GCA	CAA	CTC	TTC	TRJD2	60	19	CALGEHFLRLDPALDLTAQLFF
L04	TRDV1	GCT	CTT	GGG			ACCGTCCTA	GAAAT		CTTCAGT	ACTGGGGGATA	TAGGGGTATGGG				C	ACC	GAT	AAA	CTC	ATC	TRJD1	69	28	CALGTVLEIFSTGGYRGMGTDKLIF
L05	TRDV1	GCT	CTT	GGG			CCCCTGAG				CTGGGGG	TCTC				AC	ACC	GAT	AAA	CTC	ATC	TRJD1	45	12	CALGPLSWGSHTDKLIF
L06	TRDV1	GCT	CT	GGG	GA		CCCCGG		CCTTCC	CCGGAGGTGTACT	CTGGGGGATACG	CGATGTGTTG					CC	GAT	AAA	CTC	ATC	TRJD1	72	29	CALGDPGLPRRCTLGDTRCVADKLIF
L07	TRDV1	GCT	CTT	GGG	GAA	CT	GACGACT				ACTGGGGG					C	ACC	GAT	AAA	CTC	ATC	TRJD1	45	7	CALGELTTTGGTDKLIF
L08	TRDV1	GCT	CTT	G			CAAT				TGGGG	CATGGGACGT						T	AAA	CTC	ATC	TRJD1	36	14	CALAIGAWDVKLIF
L09	TRDV1	GCT	CTT	GGG	GAA		GGTTGTGGTC				GGGAT	TGTACGT				AC	ACC	GAT	AAA	CTC	ATC	TRJD1	51	17	CALGEGCGRDCTYTDKLIF
L10	TRDV1	GCT	CTT	GGG	GAA	C	CGGGCCCGGG				CTGGGGGAT	GCAA					ACC	GAT	AAA	CTC	ATC	TRJD1	51	14	CALGEPGPGWGMQTDKLIF
0860-1	TRDV1	GCT	CTT	GGG	GA		CCGA		TTCC	CGGACAGT							ACC	GAT	AAA	CTC	ATC	TRJD1	42	12	CALGDRFPDSTDKLIF
0860-2	TRDV1	GCT	CTT	GGG	GAA	CT	ACGA				TGGGGGATA	AT					C	GAT	AAA	CTC	ATC	TRJD1	42	6	CALGELRWGIIDKLIF
1410	TRDV1	GCT	CTT	GGG	G		TCT		TTCC			CGCCAGGT				AC	ACC	GAT	AAA	CTC	ATC	TRJD1	42	11	CALGVFPARYTDKLIF
1445	TRDV1	GCT	CTT	GGG	GA		G		CTAC	GT	TGGGGGA	AAAT					C	GAT	AAA	CTC	ATC	TRJD1	42	7	CALGELRWGKIDKLIF
888	TRDV1	GCT	CTT	GGG	G		GGGGGGG		CTTCC	CCCAAAAACCC	GGGGG	GGCCGGGCCCCCAACAGCC							CAA	CTC	TTC	TRJD2	66	37	CALGGGGFPQKPGGAGPPTAQLFF
1421	TRDV1	GCT	CTT	GGG	GAA	C	CCCA		CTTCCTAC	ATCTCAT	TGGG	ACGAT						T	AAA	CTC	ATC	TRJD1	51	16	CALGEPHFLHLIGTIKLIF
0802-1	TRDV1	GCT	CTT	GGG	GAA	CT	ATAT		CTTCCT	CCTAACTCCAATATATTATTCTTCCTACC	ACTGGGGGA	CG				AC	ACC	GAT	AAA	CTC	ATC	TRJD1	81	35	CALGELYLPPNSNILFFLPLGDDTDKLIF
0802-2	TRDV1	GCT	CTT	GGG	G		CCAGTCTT		CCTTCCT	TAAT	ACTGGGGG	CTATACAAAAAAGATTCT				AC	ACC	GAT	AAA	CTC	ATC	TRJD1	72	30	CALGASLPSLILGAIQKRFYTDKLIF
618	TRDV1	GCT	CTT	GGG	G		TACCGACTC				GGGGGATACG	CTCTAGGG				TG	ACA	GCA	CAA	CTC	TTC	TRJD2	54	17	CALGVPTRGIRSRVTAQLFF
464	TRDV1	GCT	CTT	GGG	GAA	CT	TTA		TTCC	CCTCCCCTCCCCTGGTTGG	TGGGGGAT	CCGTGG					ACC	GAT	AAA	CTC	ATC	TRJD1	69	28	CALGELYSPPLPWLVGDPWTDKLIF
																						average	57	20.15	
																						range	36-81nt	6-37nt	

Table S1.

Table includes the CDR3δ of the most dominant Vδ1^{+} clonotype for each of the liver samples that underwent NGS TCR sequencing (L01-L10), as well as single cell PCR TCR sequencing. V, D and J segment usage is identified, as well as N- and P-nucleotide (nt) addition, highlighted in red and blue respectively. CDR3 length, N/P addition and amino acid sequence for each clonotype is shown.

germline		TRDV			N/P	TRDD1	TRDD2	N/P	TRDD3	N/P			TRDJ											
	TRDV3	GCC	TTT	AG		GAAATAGT	CCTTCCTAC		ACTGGGGGATACG					AC	ACC	GAT	AAA	CTC	ATC	TRJD1				
											C	TCC	TGG	GAC	ACC	CGA	CAG	ATG	TTT	TRJD3				
																					CDR3	N/P	protein	
																					length	nts	sequence	
73R9	TRDV3	GCC	TTT	A	CGGG				ACTGGGGGATAC	CTCCCATG					CC	GAT	AAA	CTC	ATC	TRJD1	45	12	CAFTGLGDTSHADKLIF	
donor																								
2002	TRDV3	GCC	TT		CCT		CCTTCCTAC	AGTCTG	GGGGGA						C	GAT	AAA	CTC	ATC	TRJD1	42	9	CAFLLPTVWGDDKLIF	
2008	TRDV3	GCC	TT		CCAC		TCCT	CGT	ACTGGGGGAT	GA				AC	ACC	GAT	AAA	CTC	ATC	TRJD1	45	9	CAFHSSYWGMNTDKLIF	
2008	TRDV3	GCC	TTT		T				CTGGGGG	GAGGC		CC	TGG	GAC	ACC	CGA	CAG	ATG	TTT	TRJD3	42	6	CAFSGGRPWDTRQMFF	
2038	TRDV3	GCC	TTT		TGGC				CTGG	AAC						T	AAA	CTC	ATC	TRJD1	27	7	CAFWPGTKLIF	
L01	TRDV3	GCC			CTAA		CCTT	GT	ACTGGGGG	GAGCGTGAC								CTC	ATC	TRJD1	36	15	CALTLYWGERDLIF	
L02	TRDV3	GCC	T		GCCGATTTGGGT				ACTGGGGG	TC				C	ACC	GAT	AAA	CTC	ATC	TRJD1	42	14	CACRFGYWGSTDKLIF	
L03	TRDV3	GCC			CGTTCTT				ACTGGGGGATACG	GG				AC	ACC	GAT	AAA	CTC	ATC	TRJD1	42	9	CARSYWGIRDTDKLIF	
L04	TRDV3	GCC	T		CCCCGGA		CCTTCCTAC	GG						AC	ACC	GAT	AAA	CTC	ATC	TRJD1	39	9	CASPDLPTDTDKLIF	
L07	TRDV3	GCC	TTT	AG	CCAACTGCCGGTGGCT		CCTTC	ATTACC	GGGGG					AC	ACC	GAT	AAA	CTC	ATC	TRJD1	57	22	CAFSQLPVAPSLPGDTDKLIF	
L08	TRDV3	GCC	TTT	AG	TTCGGGTTA		TTCCTAC	AGGGAT	ACTGGGGGATAC	A				AC	ACC	GAT	AAA	CTC	ATC	TRJD1	60	16	CAFSSGYSYRDTGGYNTDKLIF	
L10	TRDV3	GCC	T		GCGG		CCTTCCT	GGTTAT	ACTGGGGG	CCACA				AC	ACC	GAT	AAA	CTC	ATC	TRJD1	51	15	CACGLPGYTGGHNTDKLIF	
																				average	43.9091	11.9091		
																				range	27-60nt	6-22nt		

Table S2.

Table includes the CDR3δ of the most dominant $V\delta3^{+}$ clonotype for each of the liver samples that underwent NGS TCR sequencing (L01-L10), as well as single cell PCR TCR sequencing. V, D and J segment usage is identified, as well as N- and P-nt addition, highlighted in red and blue respectively. CDR3 length, N/P nt addition and amino acid sequence for each clonotype is shown, and is also compared to the Vδ3 sequence from the 73R9 clone (Marlin, 2017).
